# Supplementary material for: Global hypo-methylation in a proportion of glioblastoma enriched for an astrocytic signature is associated with increased invasion and altered immune landscape
Source: eLife. 2022 Nov 22;11:e77335. doi: 10.7554/eLife.77335 (PMC9681209; doi:10.7554/eLife.77335)
Supplement: Figure 2—source data 1. [file elife-77335-fig2-data1.zip › Figure_2_source_data_1/Figure_2B/homerResults/motif15.similar.html]

motif15

## Information for motif15

G
T
A
C
G
C
T
A
G
C
T
A
G
C
A
T
C
G
A
T
G
C
T
A
G
C
A
T
C
G
A
T
G
C
A
T
G
C
A
T
G
C
T
A
C
G
A
T
  
Reverse Opposite:  

G
C
T
A
C
G
A
T
C
G
T
A
C
G
T
A
G
C
T
A
C
G
T
A
C
G
A
T
G
C
T
A
C
G
T
A
C
G
A
T
C
G
A
T
C
A
T
G
  

|  |  |
| --- | --- |
| p-value: | 1e-60 |
| log p-value: | -1.387e+02 |
| Information Content per bp: | 1.543 |
| Number of Target Sequences with motif | 83.0 |
| Percentage of Target Sequences with motif | 3.59% |
| Number of Background Sequences with motif | 2.0 |
| Percentage of Background Sequences with motif | 0.56% |
| Average Position of motif in Targets | 110.7 +/- 55.9bp |
| Average Position of motif in Background | 55.8 +/- 54.2bp |
| Strand Bias (log2 ratio + to - strand density) | -0.1 |
| Multiplicity (# of sites on avg that occur together) | 1.10 |
| Motif File: | file (matrix) reverse opposite |

### Similar de novo motifs found

|  |  |  |  |  |  |  |  |
| --- | --- | --- | --- | --- | --- | --- | --- |
| Rank | Match Score | Redundant Motif | P-value | log P-value | % of Targets | % of Background | Motif file |
| 1 | 0.739 | A C G T A C G T C G T A A C G T A C G T A C G T A C G T A G T C A C G T A G T C | 1e-33 | -76.307974 | 3.33% | 0.77% | motif file (matrix) |
| 2 | 0.647 | C G T A C G T A C G T A C G T A C G T A C T G A C T G A C G T A C G T A C T G A C G T A C G T A | 1e-28 | -65.512413 | 2.25% | 0.56% | motif file (matrix) |
| 3 | 0.705 | A C G T C G T A A C G T A C T G C G T A C G T A C G T A A C G T C G T A C G T A | 1e-24 | -55.610384 | 2.81% | 0.74% | motif file (matrix) |
| 4 | 0.633 | A C G T A C G T A C G T A C G T A C G T A C G T | 1e-5 | -11.851029 | 18.35% | 15.27% | motif file (matrix) |
